# Association of RASgrf1 methylation with epileptic seizures

**DOI:** 10.18632/oncotarget.18000

**Published:** 2017-05-19

**Authors:** Xiaoni Chen, Xi Peng, Liang Wang, Xinwei Fu, Ji Xiu Zhou, Binglin Zhu, Jing Luo, Xuefeng Wang, Zheng Xiao

**Affiliations:** ^1^ Department of Neurology, The First Affiliated Hospital of Chongqing Medical University, Chongqing 400016, China; ^2^ Department of Neurology, Xi'an Third Hospital, Shanxi 710000, China; ^3^ Department of Neurology, The Second Affiliated Hospital of Chongqing Medical University, Chongqing 400016, China; ^4^ Department of Neurology, The Third Hospital of Mianyang, Sichuan 621000, China

**Keywords:** RASgrf1, DNA methylation, RG108, epilepsy

## Abstract

DNA methylation, one of the mechanisms of epigenetic regulation, has been suggested to be related with epilepsy. RASgrf1 is a paternally imprinted gene and has a differentially methylated region (DMR) at the promoter that can silence gene expression. We have previously observed the down-regulation of RASgrf1 in the temporal neocortex of epilepsy patients and in the hippocampus of epileptic animals. Here, we further explored the dynamic change (1-day acute period, 10-day latent period and 45-day chronic phase) of DNA methylation and RASgrf1 expression after acute epileptic seizures in kainic acid (KA)-treated mice, and we observed the impact of N-phthalyl-L-tryptophan (RG108), a DNA methyltransferase (DNMT) inhibitor, on an acute epileptic model by polymerase chain reaction (PCR), western blotting, and bisulfite sequencing PCR (BSP). The results directly showed that the methylation of the RASgrf1 promoter gradually increased and reached a maximal level at the latent period, with subsequent suppression of RASgrf1 mRNA and protein expression levels, which reached a minimum level in the chronic phase. RG108 inhibited the increased methylation of the RASgrf1 gene, with significant inhibition occurring at the latent period, and restored RASgrf1 expression levels in the chronic phase. In addition, we demonstrated that RG108 could suppress acute epileptic seizures in KA-treated mice and epileptic discharges in 4-aminopyridine (4-AP)-treated hippocampal slices. These findings demonstrate that RASgrf1 is closely associated with epilepsy via the aberrant methylation of RASgrf1, and regulating the methylation status of relevant genes might be an intriguing topic in future research on epilepsy.

## INTRODUCTION

DNA methylation is an important epigenetic modification that can regulate gene expression. Several lines of evidence are consistent with the hypothesis that epilepsy might be associated with this epigenetic process [1–5]. Genome-wide DNA methylation changes were reported in the hippocampi after status epilepticus (SE) in kainic acid (KA)-treated mice [6]. Subsequently, a similar increase in DNA methylation was demonstrated in the hippocampi of different epileptic animals [7]. In a rat model of chronic epilepsy, a prominent increase in DNA methylation was also observed [8, 9]. Furthermore, hypermethylation of the SCN3A and GRIA2 gene promoters in animal hippocampi was identified under seizure conditions [9, 10]. In human temporal lobe epilepsy (TLE), Miller-Delaney reported that differential DNA methylation profiles of coding and non-coding genes define hippocampal sclerosis [11]; we also found DNA methylation profiles in human refractory epilepsy and some genes regulated by methylation or demethylation [12]. In addition, increased methylation of the reelin promoter [4] and increased expression of DNA methyltransferase (DNMT) 1 and 3a [13] were also found in TLE. On the other hand, valproic acid, the classical antiepileptic drug, was shown to induce DNA demethylation in cultured cells [14, 15] and in brain tissues [16]. Furthermore, the inhibition of DNMTs was able to affect excitatory neurotransmission in the hippocampus [17, 18].

RASgrf1, Ras-guanine nucleotide-releasing factor 1, is paternally imprinted and has a differentially methylated region (DMR) at the promoter that can silence gene expression. It is exclusively expressed in the neonatal brain and liver [19] and has multiple domains. RASgrf1 binds directly to the NR2B subunit of NMDA-type glutamate receptors (NMDAR) *in vivo* and *in vitro*, and it mediates neuroplasticity via the Ras-extracellular signal-regulated kinase (Ras-ERK) signaling pathway [20]. It can activate the Rac signaling pathway, which is also important for the dynamics of the cytoskeleton [21, 22]. It can bind to G proteins and transfer G protein-coupled receptor signaling [23]. Evidence has also shown that it can influence intrinsic excitability, synaptic plasticity, and neurite outgrowth [21]. Moreover, RASgrf1 (−/−) mice showed a lower seizure threshold when triggered by pentylenetetrazol and showed hyperexcitability in cultured hippocampal neurons [24]. In addition, lower expression of RASgrf1 in the temporal neocortex of epilepsy patients and in the hippocampus of epilepsy animals has been reported in our previous study [25]. Thus, we hypothesize that RASgrf1 expression and the hypermethylation of RASgrf1 might be related with epilepsy.

Here, we detected the methylation and expression of RASgrf1 at different time points in a KA mouse model. We then tested the effect of N-phthalyl-L-tryptophan (RG108), a non-nucleoside DNMT inhibitor, on the methylation and expression of RASgrf1 as well as its effect on epileptic seizure activity *in vivo* and *in vitro*.

## RESULTS

### Localization of RASgrf1 in mouse brain tissues

**Figure 1:**
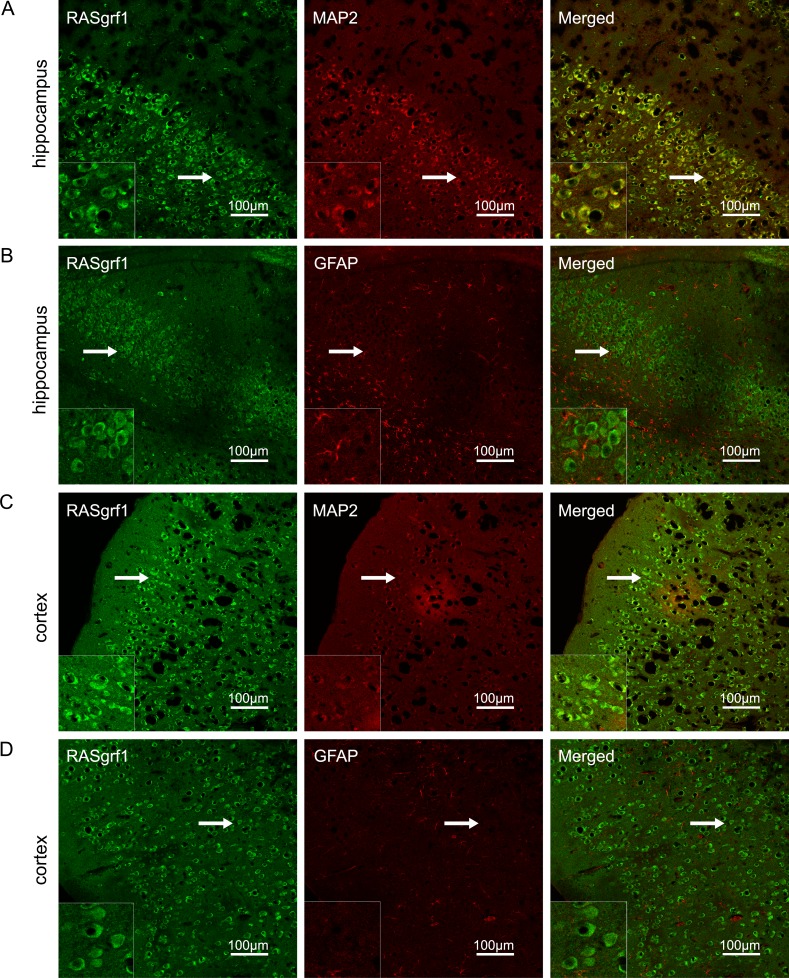
RASgrf1 double-labeling immunofluorescence in brain sections from KA-treated mice (**A**, **B**) In the CA3 region of the hippocampus, RASgrf1 (green) was co-expressed (merged) with MAP2 (red) in neurons (A) but not with GFAP (red) in astrocytes (B). (**C**, **D**) In the cortex, RASgrf1 (green) was co-expressed (merged) with MAP2 (red) in neurons (C) but not with GFAP (red) in astrocytes (D) (scale bar is 100 μm). A magnified view of the area indicated by the white arrows is located in the lower left corner (scale bar is 30 μm).

The localization of RASgrf1 in the mouse brain was estimated by double-labeling the sections with antibodies for the neuronal marker MAP2 and the astrocyte marker GFAP. RASgrf1 (green) expression colocalized with MAP2 (red) in neurons but not with GFAP (red) in astrocytes in both the hippocampus and cortex (Figure 1).

### Effect of DNA methyltransferase inhibitor on epileptic seizure activity

**Figure 2:**
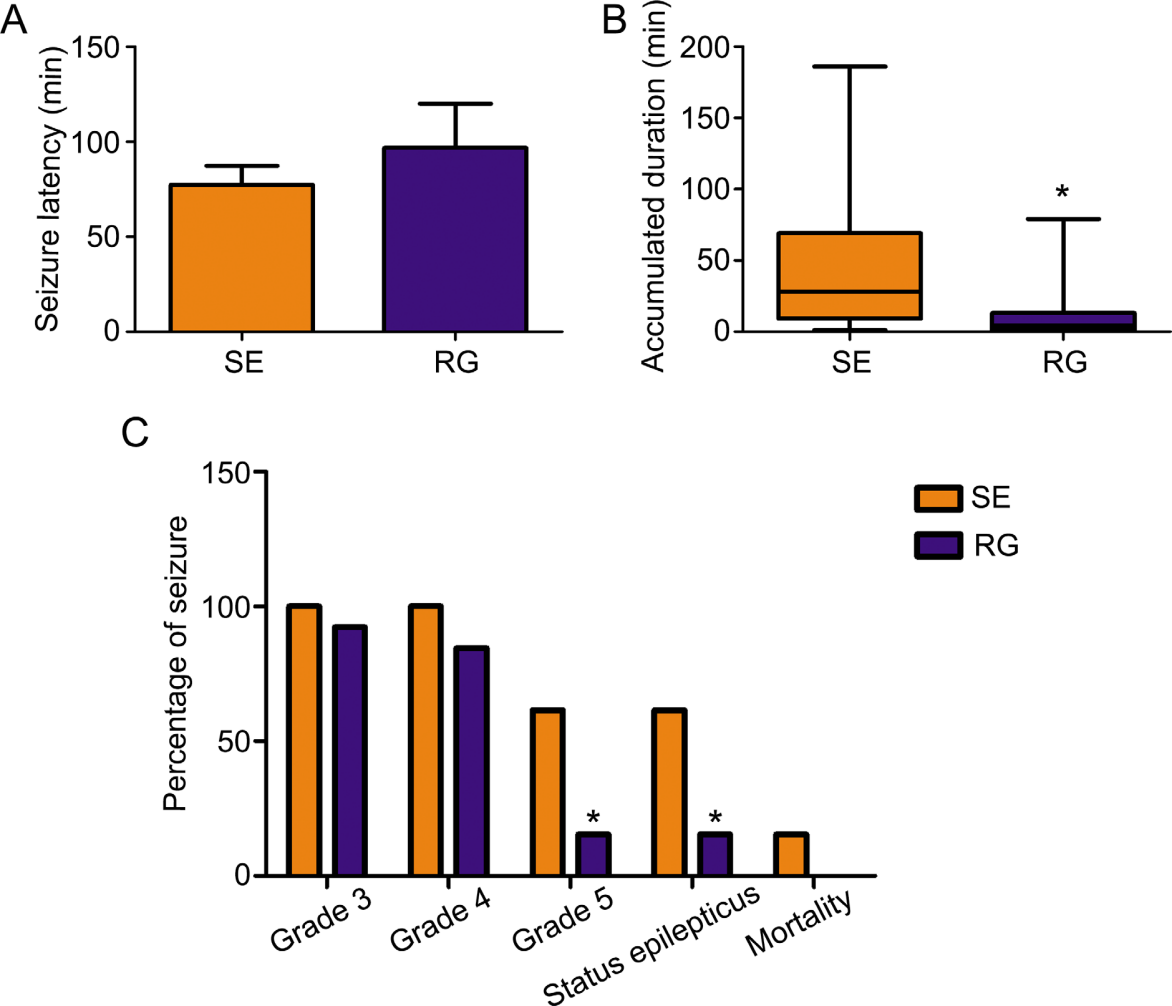
The effect of the DNMT antagonist RG108 on acute seizure behavior (**A**) After pretreatment with RG108 (RG group), seizure latency was slightly increased in KA-treated mice compared with the SE group (each group, *n* = 13). (**B**) After pretreatment with RG108 (*n* = 12), the cumulative duration of seizures (≥ grade 3) was significantly shortened. *P < 0.05, compared with the SE group (*n* = 13). (**C**) After pretreatment with RG108 (*n* = 12), the Racine score (seizure severity) was reduced in KA-treated mice, especially the rate of grade 5 seizures and status epilepticus. *P < 0.05, compared with the SE group. (*n* = 13).

**Figure 3:**
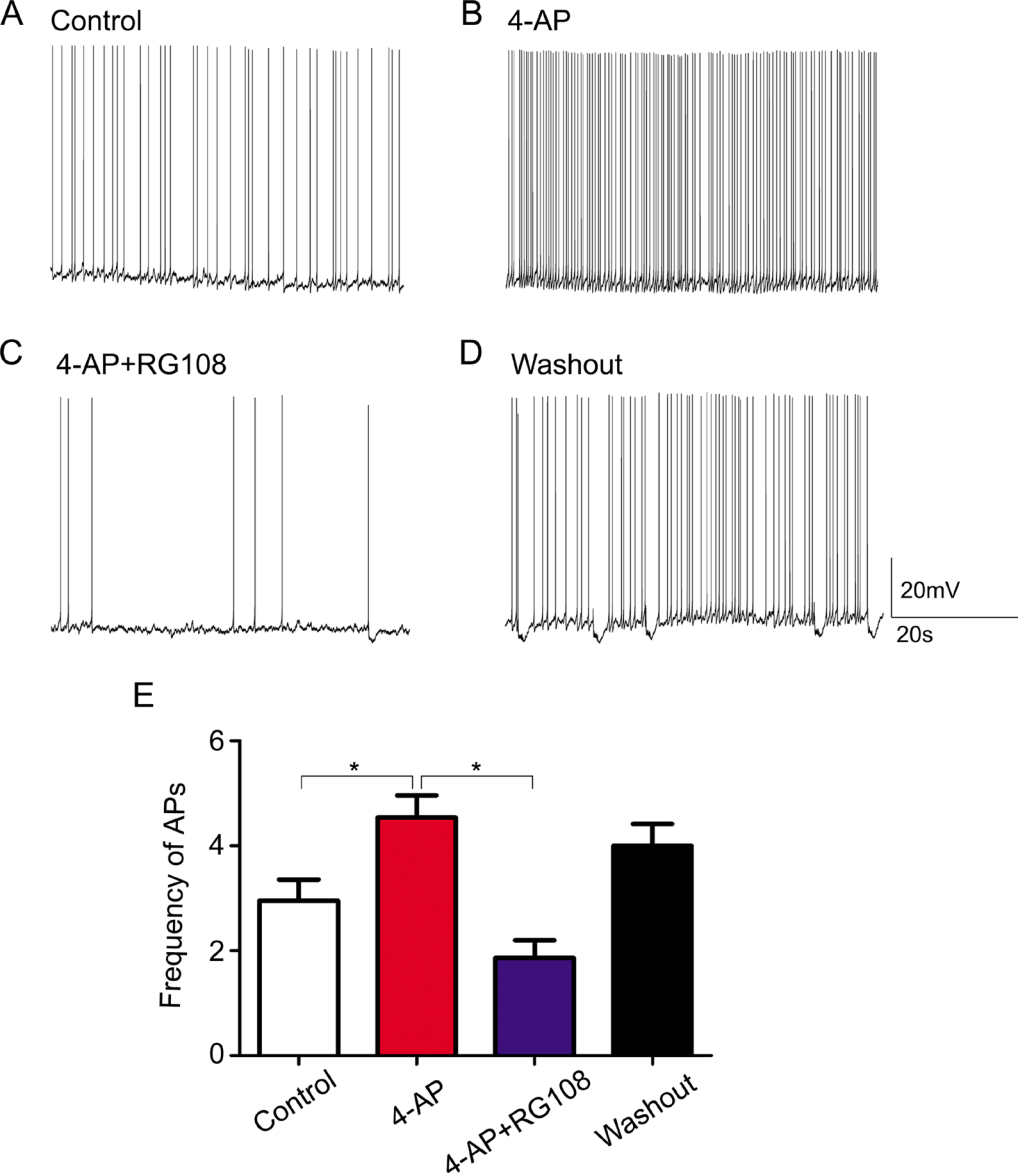
The effect of the DNMT antagonist RG108 on 4-AP medium-induced bursts of APs (**A**) The APs in hippocampal CA1 neurons of nontreated (control) brain slices. (**B**) Continuous bursts of APs occurred in the hippocampal CA1 neurons of brain slices as a result of 4-AP treatment. (**C**) After posttreatment with RG108, APs in the hippocampal CA1 neurons were significantly suppressed. (**D**) After the slices were washed with 4-AP, APs increased to some extent. (**E**) Quantitative analysis results for the AP frequencies in the different groups are shown. *P < 0.05 (*n* = 5). The membrane potential was maintained between −50 mV and −60 mV throughout the recording.

To determine whether RASgrf1 interferes with the seizure phenotype, we measured the effect of the DNA methyltransferase inhibitor RG108 on seizure activity. We observed acute epileptic seizures after 4h of KA treatment. Two mice in the SE group died of their serious SE status, but no mice died in the RG group. RG108 increased seizure latency in KA-treated mice; however, the difference in latency between the RG group and SE group was not statistically significant (96.85 ± 83.60 min in RG108, *n* = 13; 77.31 ± 36.28 min in NS, *n* = 13.) (Figure 2A). In terms of seizure severity, RG108 reduced the cumulative duration of seizures (≥ grade 3) and the Racine score (seizure severity), especially the rate of grade 5 seizures and SE in KA-treated mice, compared with the SE group, *P < 0.05 (Figure 2B and 2C). To test whether the effect of RG108 on behavioral activity was due to the inhibition of hyperexcitability, we measured spontaneous APs in the hippocampal CA1 neurons. The frequency of APs in the controls was 2.950±0.909 Hz (Figure 3A), and 4-AP infusion resulted in continuous outbursts of APs (4.540 ± 0.948 Hz) in the brain slices (Figure 3B). The frequency of spontaneous APs was significantly suppressed (1.863 ± 0.744 Hz) 10–20 min after RG108 perfusion (Figure 3C). Approximately 5 mins after the RG108 was washed off the slices, the change in AP frequency was reversed to some extent (3.997 ± 0.945 Hz) (Figure 3D). As above, RG108 was able to reduce the frequency of APs, which were mediated by 4-AP, and this effect was reversible, *P < 0.05 (*n* = 5) (Figure 3E).

### Changes in DNA methylation and expression of RASgrf1 after the onset of epileptic seizures

**Figure 4:**
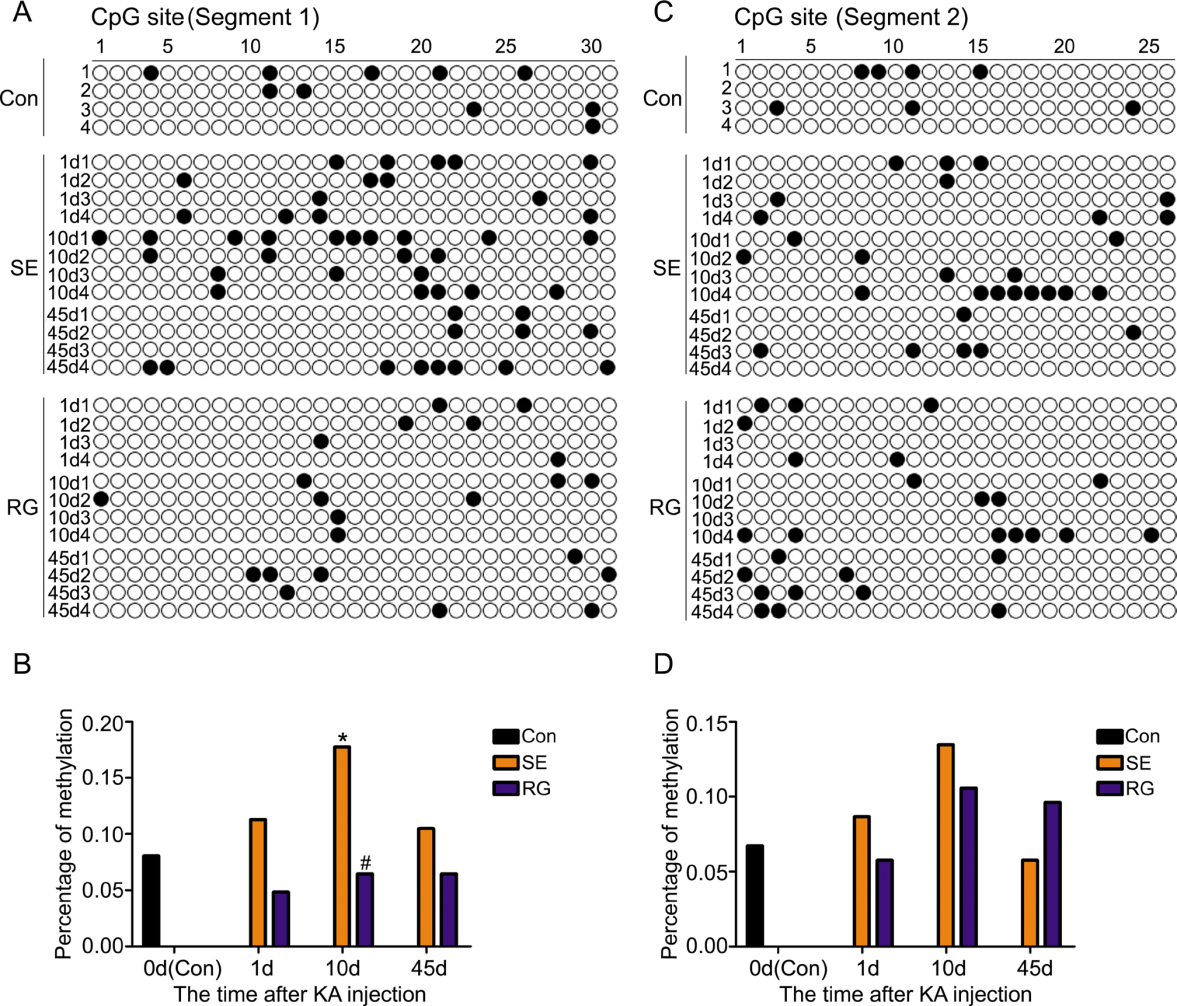
The effect of RG108 pretreatment on RASgrf1 methylation at different time points after the onset of epileptic seizures (**A**, **B**) Bisulfite sequencing shows the methylation of RASgrf1 promoter segment 1 (31 CpG sites) in control mice (control group), KA-treated mice (SE group) and RG108-pretreated mice (RG group) at different time points. There was an increase in the methylation of RASgrf1 after the onset of epileptic seizures, *P < 0.05, compared with the control group. RG108 pretreatment inhibited the increase in the methylation of segment 1, as shown 10 days after the onset of epileptic seizures, ^#^*P* < 0.05. (**C**, **D**) Bisulfite sequencing shows the methylation of RASgrf1 promoter segment 2 (26 CpG sites) in control mice (control group), KA-treated mice (SE group) and RG108-pretreated mice (RG group) at different time points. Compared with the control group, RASgrf1 methylation did not show a regular change at segment 2, nor did RG108 significantly affect segment 2 after the onset of epileptic seizures. Filled circles represent methylated CpG sites, and open circles represent unmethylated CpG sites (each group *n* = 4).

**Figure 5:**
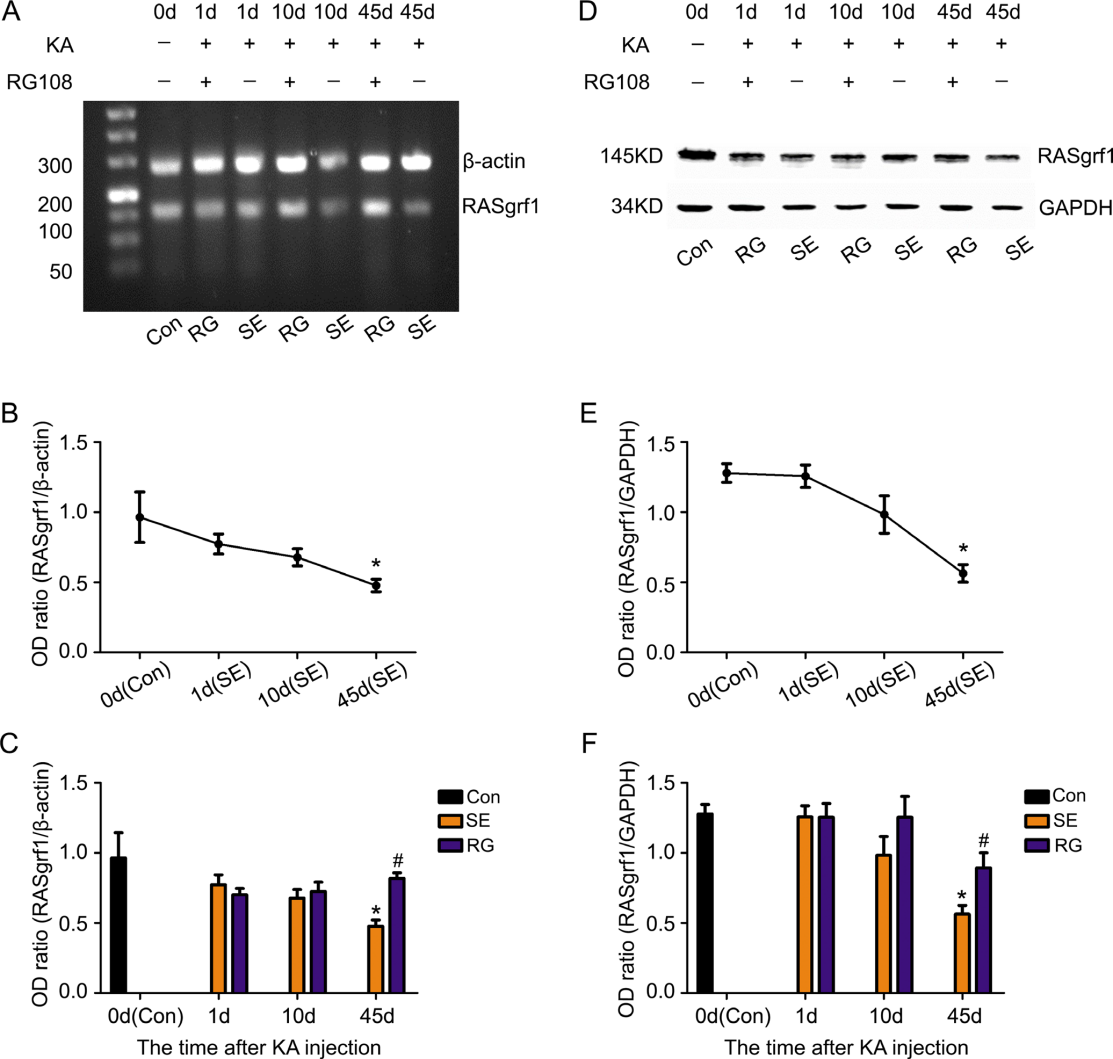
The effect of RG108 pretreatment on RASgrf1 expression at different time points after the onset of epileptic seizures Representative PCR and western blot show the hippocampus of the control group (KA-, RG108-), KA-treated(SE) group (KA+, RG108-) and RG108-pretreated (RG) group prior to KA (KA+, RG108+). (**A**, **D**) A representative PCR and western blot show RASgrf1 mRNA and protein expression in the hippocampus of the control group (lane 1), SE group (lanes 3, 5 and 7) and RG group (lanes 2, 4 and 6) at different time points. (**B**, **E**) Quantitative analyses show that the mRNA and protein expression of RASgrf1 decreased gradually after the onset of epileptic seizures and that a significant difference appeared on the 45th day. *P < 0.05, compared with the control group. (**C**, **F**) Pretreatment with RG108 reversed the decrease in RASgrf1 mRNA and protein expression, as shown 45 days after the onset of epileptic seizures, ^#^*P* < 0.05 (each group *n* = 5).

To clearly determine the RASgrf1 alterations that occur during epileptic seizures, we detected the expression and methylation of RASgrf1 at different time intervals (1-day acute period, 10-day latent period and 45-day chronic phase) after acute epileptic seizures using bisulfite sequencing PCR (BSP), RT-PCR and western blot assays. First, the results of the BSP showed that, in both segment 1 and segment 2 of the RASgrf1 promoter, the methylation of RASgrf1 in the hippocampus was increased compared with that of the control group in the latent period, but the change was only statistically significant in segment 1, *P < 0.05 (*n* = 4). Interestingly, compared with controls, segment 1 showed higher methylation, while segment 2 showed lower methylation in the chronic phase, although no significant differences were present at this stage (Figure 4). Then, to test whether RASgrf1 expression was altered in the KA-treated mice, we used PCR and western blot assays to determine the expression of RASgrf1 at different time points after acute epileptic seizures. RT-PCR results from mouse brain tissues showed that the expression of RASgrf1 mRNA was decreased at the acute period and reached a minimal level in the chronic phase. *P < 0.05, (*n* = 5) (Figure 5A and 5B). As shown by the western blot data, RASgrf1 protein levels gradually decreased and reached a minimum in the chronic phase, *P < 0.05 (*n* = 5) (Figure 5D and 5E), similar to the pattern shown by the RT-PCR data.

### RG108 inhibits DNA hypermethylation and restores RASgrf1 expression in KA-treated mice

To demonstrate the relationship between the methylation and the expression of RASgrf1 in KA-treated mice, we used KA-treated mice given a pretreatment of RG108 and compared them with mice pretreated with NS at different time points after the onset of epileptic seizures. RG108 inhibited hypermethylation in segment 1 of the RASgrf1 promoter, especially in the latent period. ^#^*P* < 0.05 (*n* = 4). However, RG108 did not appear to have a significant effect on segment 2 (Figure 4). Then, using PCR and western blot assays, we found that RG108 resulted in an obvious reversal of the decrease in RASgrf1 mRNA and protein expression in the chronic phase, ^#^*P* < 0.05 (*n* = 5) (Figure 5A, 5C, 5D and 5F).

## DISCUSSION

In our study, we demonstrated that, after acute epileptic seizures in KA-treated mice, the methylation of the RASgrf1 promoter gradually increased and reached a maximal level in the latent period, with a concurrent suppression of RASgrf1 mRNA and protein expression levels, which reached a minimum level in the chronic phase. RG108 inhibited the increased methylation of segment 1 of the RASgrf1 gene, with the most significant inhibition in the latent period. Accordingly, the RASgrf1 expression levels were restored in the chronic phase. Furthermore, in the KA mouse model and in 4-AP-treated brain slices, RG108 was able to suppress acute epileptic seizures and APs. To the best of our knowledge, this is the first demonstration of a relationship between the methylation of RASgrf1 and epileptic seizures.

Increasing evidence has suggested that DNA methylation is involved in epilepsy. In acute and chronic epilepsy models, DNA hypermethylation was observed [6–10]. A similar increase in DNA methylation has also been shown in brain tissues of TLE patients [16, 25]. Moreover, a correlation between DNA methylation status and burst frequency was demonstrated [9]. In addition, DNA methylation is frequently associated with gene silencing [26]. RASgrf1 is a paternally imprinted gene and has a CpG island-rich region at its promoter. It has been demonstrated that the methylation status of RASgrf1 directly influences RASgrf1 expression in the brain [19]. In our study, we found that the methylation of the RASgrf1 promoter gradually increased in the acute period and reached a maximal level in the latent period in the hippocampi of KA-treated mice after acute epileptic seizures, with subsequent suppression of RASgrf1 expression levels, which reached a minimum level in the chronic phase. These data show that seizure activity can lead to increased methylation of the RASgrf1 promoter and that the decreased expression of RASgrf1 after KA-induced epileptic seizures might be associated with this aberrant methylation.

RASgrf1 is highly expressed in the central nervous system (CNS) and is predominantly located in the postsynaptic densities of the mature neurons of the hippocampus and hypothalamus but not in glial cells [27, 28]. It has multiple domains, which can activate different signaling pathways. The localization and specific structure of RASgrf1 reflect its multifunctionality. Evidence has shown that it can influence intrinsic excitability, synaptic plasticity, and neurite outgrowth [21]. In RASgrf1-null mice, a higher basal synaptic activity level was recorded in the hippocampus and amygdala [29]. Tonini et al. [24] further demonstrated that RASgrf1 (−/−) mice showed increased seizure susceptibility following acute treatment with convulsant drugs and hyperexcitability in cultured hippocampal neurons. In addition, RASgrf1 (−/−) mice showed severe impairments in their amygdala-dependent long-term synaptic plasticity and in memory consolidation [29]. Studies have also shown that RASgrf1 is able to induce neurite outgrowth and that neurite outgrowth can be inhibited through the activation of Rac [21]. Additionally, a down-regulation of RASgrf1 was found in the temporal neocortex of TLE patients and in the hippocampi of chloride-pilocarpine-treated rats [25]. Therefore, we suggest that RASgrf1 is closely associated with epilepsy through the aberrant methylation of RASgrf1. However, due to its multifunctional nature, the exact mechanism of RASgrf1 in epilepsy still needs further research.

There are multiple types of DNMT inhibitors. In our study, RG108 was selected because of its long half-life (approximately 20 days) and low cell toxicity *in vivo* and *in vitro* [30, 31]. It can also reactivate epigenetically silenced genes [30]. It has been shown that mouse hippocampal slices treated with 2 h of KA exposure exhibit hypermethylation of the gria2 gene, and this effect persisted one week after the removal of the drug. RG108 was able to completely block the bursting activity and the persistent hypermethylation induced by KA [9]. In our study, the inhibition of increased methylation started on the 1st day after acute epileptic seizures, and this inhibition was significant by the 10th day when compared to the SE group. This temporal demethylation effect of RG108 could be interpreted based on the pharmacokinetics of RG108. We also found that, compared with the SE group, the RASgrf1 expression levels were slightly up-regulated on the 10th day and were significantly restored on the 45th day after acute epileptic seizures. The reason for this lies in the efficacy of RG108, which can reactivate epigenetically silenced genes [30]. Furthermore, it has been shown that DNMT inhibition (using other DNMT inhibitors for 2 h) in hippocampal neurons led to genomic DNA demethylation and a parallel decrease in the miniature EPSC (mEPSC) frequency and that this inhibition influenced neuronal excitability and network activity [18]. Meanwhile, it was demonstrated that RG108 alone had no effect on the state of DNA methylation or burst activity [9]. In our study, compared with the SE group, pretreatment with RG108 slightly increased seizure latency and significantly relieved seizure severity in KA-treated mice. RG108 also suppressed the frequency of APs in 4-AP-treated hippocampal slices. We speculate that RG108 relieved epileptic seizure activity in KA-treated mice and 4-AP-treated hippocampal slices by influencing neuronal excitability via effects on excitatory synapses. Taken together, our results indicated that RG108 could suppress acute epileptic activity by regulating neuronal excitability and could revert the increased methylation status of segment 1 of the RASgrf1 gene and restore its down-regulated expression. Is the blocked methylation status here also influenced by the relieved epileptic activity? Could RG108 interfere with epileptogenesis in chronic epilepsy? These questions still need further exploration.

In our study, compared with the controls, hypermethylation in the SE group was observed in segment 1 and segment 2 of the RASgrf1 promoter, with a significant difference in segment 1 observed during the latent period. However, in the RG group, a restored methylation status was observed in segment 1 but not in segment 2 when compared with the SE group. These results indicate that segment 1 of the RASgrf1 promoter has a more important role in silencing RASgrf1 and that RG108 mainly inhibited methylation through effects at segment 1. Therefore, segment 1 is likely to be more closely involved in epilepsy. Further study is necessary to identify the specific sites of segment 1 involved in epilepsy.

RG108, a non-nucleoside DNMT inhibitor, is a rationally designed small molecule isoform DNMT inhibitor, and its demethylating efficacy is nonspecific [30]. In our study, RG108 blocked the hypermethylation of segment 1 of the RASgrf1 promoter and suppressed acute epileptic activity, as we had predicted. However, because of the nonspecific demethylating efficacy of RG108, it is possible that RASgrf1 is not the only gene involved in the aberrant methylation status observed in epileptic activity. In spite of this, the fact that RG108 blocks epileptic seizure activity in response to KA treatment and changes the expression of RASgrf1 strongly supports the prediction that the methylation of some sites in the RASgrf1 promoter is involved in epilepsy.

In conclusion, we observed dynamic changes in RASgrf1 DNA methylation and expression in KA-treated mice. As a DNMT inhibitor, RG108 was able to reverse the abovementioned changes and suppress epileptic seizure activity *in vivo* as well as *in vitro*. Our data demonstrate that RASgrf1 is closely associated with epilepsy through aberrant methylation of RASgrf1 and that regulating the methylation status of relevant genes might be an intriguing topic for future research on epilepsy.

## MATERIALS AND METHODS

### Drug treatments and behavioral observation

All of the animal procedures in this study were approved by the Ethics Commission of Chongqing Medical University. Healthy adult male C57 mice (18-22 g) were used for experiments. Twenty-eight C57 mice, separated into two groups at random, were pretreated with RG108 (40μM) or normal saline (NS) injected into the lateral ventricle. Five days later, they were injected with KA (15 mg/kg i.p.; Sigma). The severity of behavioral seizures following KA injection was observed for 4 h and graded according to the Racine scale [32]. The onset of epileptic seizures was defined based on the mice displaying continuous seizure activity (grade 4 or 5 in the Racine scale) after KA injection. The animals were sacrificed on the 1st day, 10th day, and 45th day after the onset of epileptic seizures. All control animals were handled in the same manner as the KA-treated animals, except for KA administration.

### Tissue preparation

Some of the mice were sacrificed by decapitation after an i.p. administration of chloral hydrate (1 ml/kg). The hippocampus and neocortex were quickly dissected out on ice, frozen in liquid nitrogen, and stored at −80°C for polymerase chain reaction (PCR), western blotting and BSP. Some of the mice were anesthetized with chloral hydrate and transcardially perfused with 0.9% saline followed by 4% paraformaldehyde (4 g/100 ml) in PBS (pH 7.4). The brains were immediately removed, postfixed in 4% paraformaldehyde for 24 h, and then cryosectioned at a thickness of 10 μm for immunofluorescence.

### Double-labeling immunofluorescence

Frozen sections were fixed in an acetone solution for 30 min, incubated with 0.4% Triton for 30 min, and then incubated with 3% normal goat serum at room temperature for 1 h. Between every step, the sections were washed with PBS. For double staining, the sections were incubated with anti-RASgrf1 (1:30; Santa Cruz, USA), anti-microtubule-associated protein 2 (MAP2) (1:100; Boster, China) and anti-glial fibrillary acidic protein (GFAP) (1:100; Boster, China) at 4°C overnight. Next, the sections were washed and incubated in fluorescein isothiocyanate-conjugated goat anti-rabbit IgG (1:50; Zhongshan Golden Bridge, China) and tetramethyl rhodamine isothiocyanate-conjugated goat anti-mouse IgG (1:50; Zhongshan Golden Bridge, China) in the dark for 60 min at room temperature. Fluorescent images were collected by laser scanning confocal microscopy (Leica, Germany) using an Olympus IX 70 inverted microscope (Olympus, Japan).

### Western blot analysis

Protein extracts from brain tissues were separated by SDS-PAGE and transferred to nitrocellulose membranes. After being blocked, the membranes were incubated at 4°C overnight with the following primary antibodies: anti-RASgrf1 (1:400; Santa Cruz, USA) and anti-GAPDH (1:1000; Santa Cruz, USA). The membranes were washed and incubated with a horseradish peroxidase-conjugated secondary antibody (1:4000, Zhongshan Golden Bridge, China) for 1.5 h at 37°C. The protein bands were visualized with an enhanced chemiluminescence substrate (Pierce, USA) and scanned (Bio-Rad Laboratories). The optical density (OD) of these bands was quantified using Quantity One software (Bio-Rad Laboratories).

### RNA isolation, reverse transcription and PCR

Total RNA was extracted from brain tissues using RNAiso Plus (TaKaRa) and reverse transcribed using a First Strand cDNA Synthesis Kit (TaKaRa) according to the manufacturer's instructions. The amplifying conditions were as follows: heating at 94°C for 5 min, followed by 30 cycles of denaturation at 94°C for 30 s, annealing at 56°C for 30 s, and extension at 72°C for 30 s, followed by a final extension at 72°C for 5 min and 4°C for 10 min. β-Actin was used as the control for the reaction. The PCR products were confirmed by electrophoresis on 2% agarose gel. The primers used are summarized in Table 1.

**Table 1:** PCR primer sequences (5′→3′)

Gene	primers	Length (bp)
β-actin	Forward GAGACCTTCAACACCCCAGC Reverse ATGTCACGCACGATTTCCC	263
RASgrf1	Forward GCACACCCAGGACTTTGATAC Reverse TGCTATGTTGTTCAGTTGTTTCTTC	161

### Gene segment selection and BSP

Currently, BSP is the gold standard for directly detecting the methylation status of genes. Mouse hippocampal tissue (10 mg) was used for genomic DNA extraction with the phenol–chloroform method, and the DNA was treated with the bisulfite-modifying DNA method.

**Figure 6:**
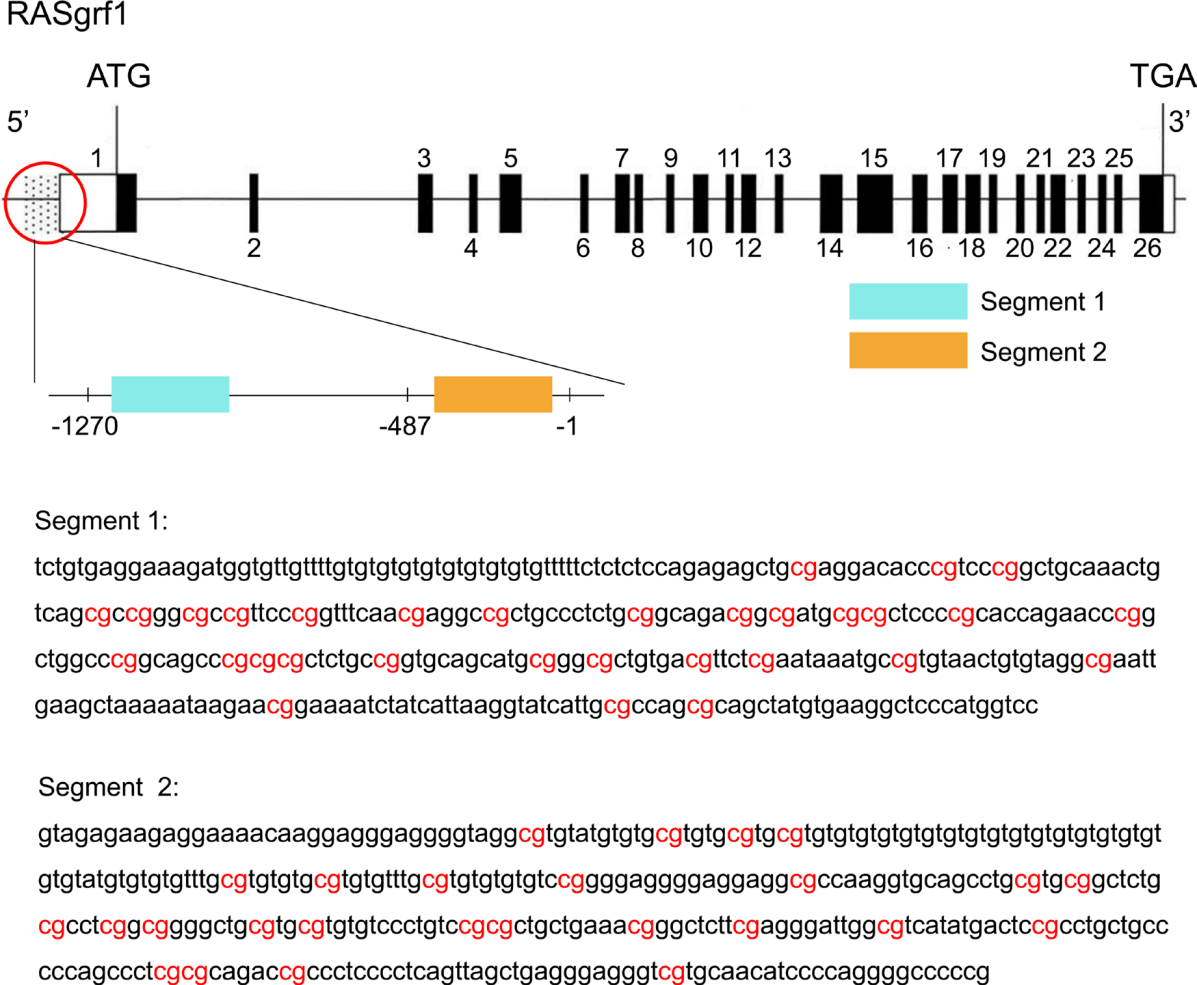
Genomic structure of RASgrf1 and sequencing of its methylated segments The exons of RASgrf1 are represented as black boxes with the numbers above. Open boxes represent either 5′ or 3′ untranslated regions (UTRs). The dotted part represents the promoter of RASgrf1, and a magnified view of the promoter area indicated by the red circle is located under the circle. Segment 1 (−1210/−862) and segment 2 (−395/−49) (the transcription start site is defined as +1) DNA sequences of the 5′-end of the RASgrf1 gene are displayed in the figure. Highlighted in red: the CpGs analyzed. The figure were assembled using data from the UCSC genome browser (http://genome.ucsc.edu/).

The promoter for the RASgrf1 gene is generally consider to be a 2000 bp sequence upstream of the transcription start site. The -487/−1 and -1270/−487 promoter regions containing the AP-1 or Oct1 binding sites were initially determined to enhance RASgrf1 expression. Therefore, we selected two CpG-rich segments from the above two regions for methylation sequencing (Figure 6). The primer for RASgrf1 was designed in MethPrimer (http://www.urogene.org/cgi-bin/methprimer/methprimer.cgi) and synthesized by the Sangon Company (Shanghai, China). The primers used are summarized in Table 2. Three microliters of the bisulfite-modified DNA from each sample was subjected to PCR analysis in a 50 μL volume (10 × PCR buffer (with Mg^2+^), 2.0 mmol/L MgCl_2_, 2.5 mmol/L dNTP, 1 mmol/L primer, and 5 U/μl Taq enzyme). The amplifying conditions were as follows: preheating at 98°C for 4 min, and then 20 cycles of denaturation at 94°C for 45 s, annealing at 66°C for 45 s, and extension at 72°C for 60 s, with the annealing temperature decreasing by 5°C each cycle, followed by 20 cycles of 94°C for 45 s, 56°C for 45 s, and 72°C for 60 s and a final extension of 8 min at 72°C. Amplified bisulfate PCR products were purified and recovered using a SanPre Column PCR Purification Kit and then subcloned into the pUC18-T vector system (Sangon) according to the manufacturer's instructions. DNA sequencing was performed on ten individual clones (Sangon). The PCR products were confirmed by agarose gel electrophoresis.

**Table 2:** BSP primer sequences (5′→3′)

RASgrf1	primers	Length (bp)
Segment 1	Forward TTTGTGAGGAAAGATGGTGTTG Reverse AAACCATAAAAACCTTCACATAACTAC	349
Segment 2	Forward GTAGAGAAGAGGAAAATAAGGAGG Reverse CGAAAACCCCTAAAAATATTACAC	347

### Slice preparation and whole-cell recording

Brain slices (350 mm) were prepared from 2-week-old Sprague-Dawley rats. The rats were decapitated, and the brains were rapidly removed and placed in ice-cold oxygenated (95% O_2_-5% CO_2_) cutting solution. The slices were perfused with artificial cerebral spinal fluid (ACSF), constantly bubbled with 95% O_2_ and 5% CO_2_, and were incubated at 25°C for 30 min and then at room temperature. Intracellular whole-cell patch-clamp recordings were taken using a MultiClamp 700B amplifier (Axon, USA) and pClamp 9.2 software (Molecular Devices, Sunnyvale, CA, USA) under an inverted microscope (Olympus, U-AN-2, Japan). Glass micropipette electrodes (Sutter) filled with intracellular solution were used to record action potentials (APs). Intracellular recordings were generated from the pyramidal cell layer of CA1 using whole-cell current-clamp techniques. 4-Aminopyridine (4-AP) was diluted to a stock concentration of 100 μM. RG108 was diluted to a stock concentration of 40 μM. All of the fluid formulas and experimental conditions have been reported in a previous article [33].

### Statistical analysis

The data were analyzed using SPSS 21.0 software and reported as the means ± SD. Statistical significance was assessed using Student's *t*-tests, Student's paired-sample *t*-tests or the Mann–Whitney *U* test for pairwise group comparisons. Comparisons of more than two groups were tested using one-way ANOVA. The chi-squared test was used to analyze the enumeration data. The statistical significance was set at *P* < 0.05.
